# Designing and Implementing a Curriculum to Support Health Equity Research Leaders: The Interdisciplinary Research Leaders Experience

**DOI:** 10.3389/fpubh.2022.876847

**Published:** 2022-05-13

**Authors:** Sarah E. Gollust, Kathleen T. Call, J. Robin Moon, Bonnie Cluxton, Zinzi Bailey

**Affiliations:** ^1^Division of Health Policy and Management, University of Minnesota School of Public Health, Minneapolis, MN, United States; ^2^Department of Health Policy and Management, CUNY Graduate School of Public Health and Health Policy, New York, NY, United States; ^3^AcademyHealth, Washington, DC, United States; ^4^Divisions of Medical Oncology and Epidemiology, Department of Medicine, Miller School of Medicine, University of Miami, Coral Gables, FL, United States

**Keywords:** health equity, curriculum, leadership, education, policy

## Abstract

Health inequities in the United States are well-documented. However, research that is focused on solutions, rather than just describing the problem, and research that is designed explicitly to inform needed policy and practice change, is still too rare. The Robert Wood Johnson Foundation *Interdisciplinary Research Leaders* (IRL) program launched in 2016 with the goal of filling this gap: to generate community-engaged research to catalyze policy action in communities, while promoting leadership among researchers and community partners. In this paper, we describe the creation and implementation of a curriculum for IRL program participants over the first 5 years of the program. The curriculum—spanning domains of leadership, policy, communication, community engagement, and research methodologies—was designed to cultivate leaders who use research evidence in their efforts to promote change to advance health equity in their communities. The curriculum components implemented by IRL might be applied to other educational programs or fellowships to amplify and accelerate the growth of leaders nationwide who can use research and action to respond to grave and ongoing threats to community health.

## Introduction: Background and Rationale

The United States (U.S.) has extreme (and in some cases, worsening) health inequities across various dimensions, including race, ethnicity, social class, rurality, sexual orientation, and gender identity (1). For the past 20 years, research from multiple disciplines has increasingly documented these health differentials due to the social determinants of health equity (2)—including disparities in health behaviors like smoking, health outcomes like cardiovascular disease or mental illness, and differences in the availability of social resources known to promote or inhibit health (3). The life course perspective on cumulative as well as intergenerational effects of these dimensions on health has illuminated the importance of health disparities in the earliest stage of life (4). Recent data demonstrate that the COVID-19 pandemic has contributed to reductions of life expectancy in the U.S and worsening racial disparities in life expectancy (5). In short, there is now an abundance of evidence of the problem of health disparities and the social determinants of health that produce those disparities.

However, there remain many challenges related to the current evidence-base from health disparities research. One challenge is that there is comparatively less research evidence on the solutions, as opposed to only documenting problems. Further, what research there is on interventions is often on clinical interventions, as opposed to interventions based in communities or systems (6). Health disparities research in the past has often focused on individual-level risk factors (along with corresponding behavior change solutions), and only more recently has examined system-level causes (along with corresponding system-level, legal, policy, and political solutions) (7–9). Further, the research that exists is often not effectively translated into the hands of those with the power to make change, whether policymakers, community organizers, or practice leaders in non-governmental organizations, nor is it necessarily communicated back to affected communities (10). Research has often been used to further advantage researchers and institutions conducting the research, rather than being led by and used to increase the power of communities that have the most at stake with the results of the research. Previous research training programs have produced strong evidence of benefit to researchers (i.e., publications, promotions, etc) and their research institutions, but less evidence of benefit to communities and specific policy actions (11). In short, to have maximum impact on advancing health equity, research needs to be done differently.

The Robert Wood Johnson Foundation *Interdisciplinary Research Leaders* (IRL) program was borne with the audacious goal of building leaders who can disrupt and respond to these challenges. The program was designed with a few key structural features. First, IRL fellows enter the program in teams of three: two interdisciplinary researchers work with one community leader who is accountable to and represents the community, is an expert in the community conditions, and is connected to other local leaders who are well-poised to act on the research findings. Second, each member of the trio is supposed to be an equal partner and equal recipient of the fellowship program, curriculum, and resources. However, while no team member is below any other in stature in the idealized program vision, we recognize that power differentials across team members often emerge (a challenge discussed more below).

Third, the fellows are part of a carefully-selected cohort of diverse but likeminded teams working on similar health equity issues across the U.S. Specifically, the call for application is explicitly open only to one or two specific themes each year, and these themes are purposefully chosen to emphasize structural, environmental, or policy-oriented health equity issues, not individual behavioral issues. The thematic foci for the first five cohorts were: 1) Early childhood / Housing and community development; 2) Individual and community resilience / youth development approaches to prevent violence; 3) Social determinants of rural health and rural health care; 4) Community development and health / clinical practice, social services, and health; and, 5) Community environment and health / families and child health. Within each cohort's thematic area, applicants working on solutions beyond the individual level were prioritized. This cohort structure maximizes teams' opportunities to connect and overcome challenges and share leadership struggles, and provides a community of practice and support for research partners and community partners alike to share challenges and strategies. Fellows are selected to maximize multiple forms of diversity across the cohort (e.g., geographic, disciplinary, topic area, research and leadership experience, and the racial, ethnic, and socioeconomic diversity of the populations proposed to be engaged). The thematic cohort model also increases the likelihood that project results are synergistic and drive measurable progress in each health equity-related topic area, and facilitates a broad network of champions advocating for evidence-based solutions. As a testament to the success of the thematic cohort model, members of the first cohort (2016-2019) published collected volumes and journal special issues discussing process and findings from the first thematic areas: early childhood health (12) and housing and health (13, 14).

The IRL program consists of a research project, a focused curriculum designed to introduce and deepen leadership skills and capacities, and practical experience in translating research results to achieve policy or practice impact. Fellows in the program receive funds for their participation in program activities ($25,000 per year) and each team receives a moderate research grant (around $100,000). The overarching programmatic goal is to produce leaders in conducting innovative, rigorous, action-oriented, team-based research that can be used to stimulate action to build a culture of health in the U.S. The goal of the curriculum is thus to cultivate research and community leaders, with an emphasis on how leaders can use research evidence in their efforts to promote change to advance health equity. While most research funders (including federal government funders and foundations) simply provide research grants and request intermittent reports and final publications, the IRL program is different —as a leadership program *and* a research funding program. One major source of difference is the focused curriculum that connects all grantees. This curriculum provides the foundational program experience that transforms a research grant into a catalytic program to spur leaders toward action. The objective of this paper is to describe the creation and implementation of this curriculum. It is our expectation that components of this curriculum can be applied to other educational programs or fellowships to amplify and accelerate the growth of leaders nationwide who can use research and action to advance health equity.

## Theoretical Underpinnings: Pedagogical Frameworks and Principles

The curriculum was designed with a few underlying theoretical constructs in mind. First, the IRL program relies upon a foundation of decades of work in community-based participatory research (CPBR), providing teams with an established framework for thinking about how to co-construct research questions, research design, and processes to deeply reflect community needs and values (15, 16). However, the program has expanded beyond some key tenets of CBPR given the specific structure of the IRL research team, consisting of two researchers and one community leader, which requires immediate—and consistent—attention to what it means to collaborate equitably (17).

Second, to make change in communities, IRL teams need to understand processes of policy change—including the formal policy process (in both legislative settings and organizational settings) and community-power building methods and processes. By learning about the methods of community organizing, fellows learn tangible skills in mapping power in communities, the importance of relationship-building to social movements, and how to identify and understand multiple stakeholders' interests for change in order to move people toward action (18–21).

Third, theories from dissemination science point toward the importance of developing pro-active and early plans for dissemination, and to do this in relationship with stakeholders who can help not only lead research questions but also center community in the types of dissemination outputs and outcomes that are important for the ultimate end-users of the research (22, 23). This dissemination science evidence-base strongly reinforces developing dissemination plans early in the research project and designing communication outputs that are multiple and tailored for varied users (including non-peer-reviewed research briefs, social media, oral communication, in-person testimony, and peer-reviewed publications and presentations) (24–27). Communication is core to the program—not only to disseminate research results, but also for fellows to raise their own voices to reshape the narratives around who counts as a researcher, who produces cutting-edge research, which environments foster equitable community engagement and leadership, and what a healthy U.S. would look like. By investing in fellows' voices, the IRL program aims to shift broader cultural and social narratives about health and racial equity, by elevating a structural lens on health issues which are too often framed in public discourse as individualized and oriented around health care and personal behaviors (28–30). Further, the dedicated—and expanding—curriculum on structural racism and health provides the conceptual scaffolding for fellows to use their voices, with common language and orientation, to explain the impact of racism, not race, as the driver of inequality (31).

Fourth, the curriculum communicates the strong perspective that for research to inform practice and policy change, it must be done with credibility and transparency. Research questions must be feasible to answer, the community must be genuinely interested in the answer (even if the answer does not support the key hypothesis), and the work must be done with integrity and scientific rigor. The program also values developing a shared language for non-researcher leaders around the goals and research methods, honoring expertise in the community alongside research expertise. Research produced in the program should identify actionable and precise answers to community, practice, and policy-relevant questions.

Last, and critically, leaders must interrogate their own personal, professional, and institutional perspectives around Equity, Diversity, Inclusion (EDI) and Anti-Racism. Leaders in health equity must not only understand their own personal stake in advancing equity and the specific rootedness of racism as a key structural determinant of inequity, but they also must learn how they can engage in anti-racist actions as educators, researchers, community leaders, and advocates for organizational and systemic change (32). Although not the only form of social marginalization, racial inequities are embedded in American government and institutional structures and are inextricably tied to all other forms of oppression. As such, racism is at the center of all pathways to achieving health equity, including access to jobs, housing, education, income, and health care, while interacting with other systems of oppression (31). While conducting research to advance health equity was core to the program from the start, the dedicated focus on structural racism and anti-racism emerged in the first few years of the program in response to the social and political environment as well as explicit calls from fellows. During this transition period, the IRL program also sought to diversify the racial composition of its leadership and form a fellow- and alumni-driven Equity, Diversity, and Inclusion Task Force to infuse these principles and measures of accountability within all aspects of the program.

Each of these theoretical constructs contributes to the four pillars of the curriculum, described below and in Table 1. The four curriculum pillars and the learning objectives are:

- Community Change Leadership (CCL)—Understand issues facing the community and how to build and lead movements to create change that promote health and racial equity.
- Policy and Communication (PC)—Understand the policy-making process, identify relevant stakeholders, and communicate research outcomes to achieve policy objectives.
- Collaboration and Community Engagement (CCE)—Facilitate effective and equitable research-community partnerships across diverse disciplines and sectors.
- Credible and Transparent Research (CTR)—Apply scientifically sound methodologies to answer relevant community-oriented research questions.

**Table 1:** Pillars of the IRL curriculum (2016–2021).

**Curriculum Pillar**	**Example program elements**
Community change leadership	Community organizing trainings; equity diversity and inclusion trainings; resilience coaching
Policy and communication	Dissemination planning; messaging and communications training; media training; data visualization; op-ed writing support
Collaboration and community engagement	Equitable collaboration partnership planning; community-based participatory action principles; team coaching
Credible and transparent research	Research methods; focus groups; research workshops (e.g., research questions, logic models) individualized research support and mentoring

While the above pillars and associated theoretical principles comprise the conceptual grounding that informs the *substance* of the curriculum, the program also applies adult learning principles to the *process* of how fellows engage with the curriculum (33). The curriculum relies on adult learning principles as well as the active engagement and rapid evaluation of the curriculum users themselves—IRL fellows—to create a flexible and adaptable program experience. Table 2 describes key adult learning principles and how the program responds to them in its design and implementation. Considered collectively, the curriculum aims to activate fellows so that their future trajectory as researchers and community leaders is action-oriented and change driven. As such, the program not only invests in fellows during the program, but also into their experience as alumni, by engaging them in webinars and meetings to maintain their networks into the future.

**Table 2:** Adult learning principles incorporated into IRL program curriculum.

**Principle**	**Implementation**
Adult learners want to be involved / collaborators in learning	• Solicit ideas for webinars and mini-courses annually and encourage continuous feedback • Incorporate fellow-led and fellow-selected webinar topics throughout the curriculum
Adult learners want to know why they are learning what they are learning	• Consistently communicate learning objectives and rationale for curriculum, including in in-person meeting agendas
Adult learners want flexibility and self-direction	• Online course content is organized into small modules that are labeled so learners can self-navigate • Consistently communicate that fellows draw on what they wish, with clear signals of which courses and readings/resources are to be considered supplemental/optional
Adult learners have a large foundation of knowledge and experience	• Communicate the IRL program as a “learning community” and emphasize co-learning and for fellows to contribute their expertise for their cohort
Adult learners are practical and goal-oriented	• Require only deliverables with practical utility (i.e., dissemination plan, collaboration agreement for team, research brief)
Adult learners demand respect	• Develop community-accepted “ground rules” to communicate expectations of respect, mutual learning, and growth for all program participants, known as the IRL Community Agreements for Learning and Practice

## Program Experience: Organization, Learning Environment, and Community of Practice

The IRL program is managed by a National Program Center (henceforth called NPC) based at the University of Minnesota School of Public Health. By design, the NPC consists of faculty, staff, partners and consultants from both academic and non-academic (i.e., community organizing, community development, public health practice) backgrounds. The IRL program is one of four national leadership programs funded by RWJF which all seek to make progress toward building health equity and a national Culture of Health, but each program is independently managed, and the curricula are distinct and created by the NPC. The programs do have some shared curriculum through cross-program meetings, described below. The IRL NPC not only plans and delivers curriculum through meetings and webinars (the curriculum components described below) but also provides a “high touch” experience through abundant personal interaction with fellows.

**Figure 1:**
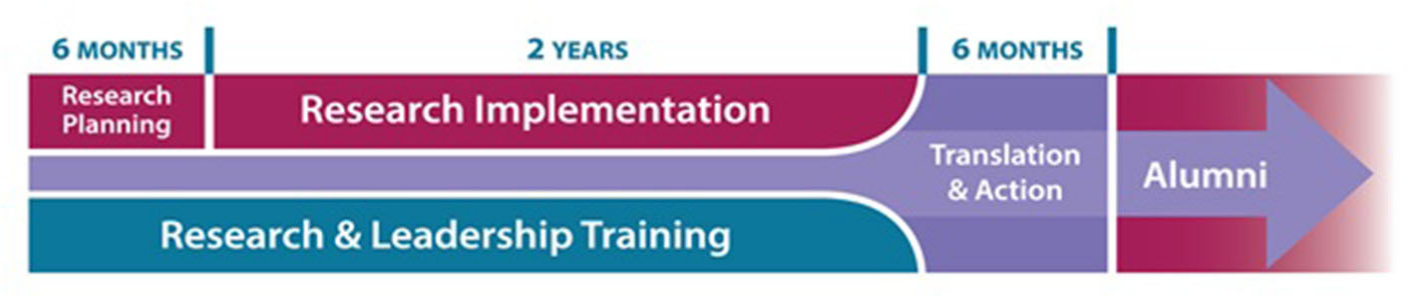
Schema depicting the IRL program fellowship years.

While the cornerstone of the IRL experience is the research project that all teams plan, implement, and disseminate, the IRL curriculum supports teams in doing this work throughout the life of the project. There is a dedicated focus on *translation and action* throughout the program under the theoretical motivation described earlier that research must be disseminated and framed to resonate with the community leaders, organizations, and policymakers who drive change (see Figure 1). Per previously noted theories of policy dissemination (24), research teams that wait to disseminate their work only when it is complete—and miss the opportunity to strategize and build relationships with journalists, community organizers, advocacy organizations, policymakers and other research intermediaries—are less likely to see action result from their research.

The IRL curriculum is delivered through a combination of in-person experiences, online courses, and the creation and maintenance of a learning community through regular webinars which allow for within- and cross-cohort learning and professional development. (In 2020–2021, the in-person meetings were paused because of the COVID-19 pandemic). We integrate the content across the in-person experiences, the synchronous webinars, and the online Mini-Courses, as much as possible.

### In-person Experiences

The original program design called for eight in-person meetings spanning the 3 years of the fellowship. A “Minnesota Leadership Meeting” occurs every fall, held in Minnesota. A “Communication and Policy Workshop” occurs every spring in the first and third years of the fellowship, and an annual Leadership Institute (convening all four national leadership programs funded by RWJF) occurred each year to total eight meetings (the latter meeting ended in 2020 as a result of the COVID-19 pandemic). Each meeting allows for dedicated attention to particular curriculum components. For instance, the fall meeting focuses around the Community Change Leadership pillar, and the highlights are interactive sessions that highlight community organizing and community power building activities as well as experiential opportunities to learn from and interact with organizers who have used research and the methods of community organizing to achieve successful action in their communities. The organizing curriculum is based upon the premise that centering the power and voice among the communities who are most affected by inequities is an important mechanism toward advancing health equity (18, 34).

The spring meeting focuses on the Policy and Communication pillar, and includes discussions of policy agenda-setting, framing, use of research evidence to inform policy, and—for third year fellows—practice creating research briefs or other dissemination outcomes from their project. The other in-person meetings offer flexible time for sessions (often fellow-led) and importantly, the networking and relationship-building opportunities that are harder to create and foster in a virtual setting.

### Weekly Webinars

A hallmark of the IRL experience is a regular webinar series held roughly three times per month for each cohort throughout the fellowship years. The weekly webinars have a few objectives. First, they facilitate an opportunity for connection (mainly within-cohort, and occasionally cross-cohort), which is essential for a hybrid program to establish relationships among fellows. Second, they provide a synchronously delivered curriculum space for speakers and discussion to amplify content and interaction from the Mini-Courses (see below). Third, they allow fellows the opportunity for engagement with leaders in community-engaged action-oriented research who are invited to share their stories, advice, or research. Finally, the webinars are the key venue in which teams share their ongoing work with their cohort. Specifically, through regular Work-in-Progress (WIP) webinars each year of the program, teams share their research planning, process, methods, community engagement, challenges or obstacles, key findings, and dissemination opportunities with IRL staff and leadership as well as fellows. The WIP sessions also provide a skills-based opportunity for fellows to both ask for and receive feedback as well as to learn about disciplinary methods and inquiries outside of their own training. In 2020 and beyond, we aimed to make the webinars more interactive (in part because of pivoting to an entirely virtual program) by creating opportunities for pairs of teams to present and/or for teams to integrate more interaction and community learning (i.e., through break-out rooms and discussion questions) instead of just didactic presentation and feedback. In addition to the WIP webinars, we invite fellows to share topics of interest to them and their cohort and design the series to fit their needs and expectations as ascertained via survey and intake data from all cohorts.

### Mini-Courses

Some content is best conveyed in self-paced video courses, outside of the webinar series. Over the first 5 years of the program, the IRL NPC has built a library of 15 video-based “Mini-Courses” that are between 60 minutes and 3 hours long, consisting of video content plus a curated list of relevant readings and resources (Table 3). The IRL NPC has selected instructors to create these courses (and has also involved current fellows and alumni as instructors and lecturers) on topics that were identified as critical to IRL based on the “Theoretical Underpinnings” (above) or by fellow request through regular surveys and feedback. While a few of the courses are “required,” have associated deliverables as part of them, and are delivered in a specific sequence, most of them are optional, and fellows can view them at their leisure during the program. Each course tracks back to one of the four curriculum pillars (Table 3). The main rationale for having required mini-courses is so that all teams have a shared understanding of key constructs considered important to the goals of IRL, and to build shared vocabulary and learning across fellows who come from very different backgrounds and experiences. The required mini-courses also provide a sequence and anchor for some required IRL deliverables. For instance, fellows in the Equitable Collaboration course in the first few months of the first year complete a first draft of their teams' Collaboration Agreement. Fellows in the spring of their first year take Planning for Dissemination and Use of Evidence in Policymaking and complete the first draft of a dissemination plan. These documents provide teams with a grounding on navigating an equitable collaboration and on planning for dissemination, core principles of the IRL program curriculum.

**Table 3:** Video-Based mini-courses as part of the IRL curriculum.

**Course name**	**Sequence and timing**	**Curriculum pillar**
**Required/ Strongly recommended courses**		
Equitable collaboration: core concepts, tools and approaches	Year 1 through fall Year 2	CCE
Community-Engaged research: rationale, principles, steps and strategies	Mainly year 1, some modules in year 2 & 3	CCE / CTR
Advancing health equity in research	Fall, year 1	CCL / CTR
Research literacy and introduction to causal inference	Fall, year 1	CTR
Planning for dissemination and use of evidence in policymaking	Spring, year 1	PC
Data visualization	Fall year 2	PC / CTR
Media training	Fall year 2	
Introduction to health impact assessment	Fall year 2	PC / CTR
**Optional courses available to fellows**		
Focus group interviewing	Spring year 1	CTR
Social media research dissemination	Summer Year 1, after DC meeting	PC
Implementation science	Summer year 1, after DC meeting	CTR
Program evaluation: a tool for community change	Spring year 2	CTR
Documenting community engagement in promotion and tenure	Spring year 2	CCE
The evolution of federal Native American policy	Anytime	CCL
A life course perspective on partnership sustainability	Fall year 3	CCE

*CCL, community change leadership; PC, policy and communication; CCE, collaboration and community engagement; CTR, credible and transparent research*.

### Other Curriculum Elements

As the program evolved, the NPC team realized that in addition to the meetings, webinars, and mini-courses, health equity leaders can also benefit from other more individualized experiences to deepen their leadership capacity, research skills, and ongoing work to create social change. These program experiences include resilience coaching, dissemination coaching, team coaching, and research support.

Fellows have been offered the opportunity to participate in resilience coaching sessions since 2020. Resilience coaching is a combination of leadership coaching and therapeutic emotional support, motivated by the fact that rates of burnout and stress are higher for people working in social justice related fields (35–37). Dissemination coaching was a feature of the program since its founding. Through contracts with various experts in dissemination, we have offered teams the opportunity to have focused coaching on writing and public outreach and engagement, such as writing Op-Eds, working with media, or pitching stories to journalists. In addition, team coaching has been made available to teams struggling with equitable collaborations on an *ad hoc* basis since 2017. Interdisciplinary community-research partnerships are challenging, and part of the core program work is navigating conflict of priorities, budgeting, power, and project management within teams. Last, the NPC developed an individualized approach to providing IRL research teams with specific support on research projects through a dedicated staff within the NPC that engages regularly with teams on their needs—including specialized access to mentors, workshops on logic models and research questions, research design support, and statistical analysis support.

## Assessment

While presentation of evaluation data itself is beyond the scope of this manuscript, below we outline the various types of data the IRL NPC has collected that has allowed the team to continuously improve the quality and relevance of the curriculum for program participants. First, annually (and just before the cohort begins their first year), fellows take a quantitative survey self-assessing their competencies through Likert-scale items encompassing core domains across the four curriculum pillars: Community Change Leadership, Policy and Communication, Collaboration and Community Engagement, and Credible and Transparent Research. These quantitative metrics allow us to track growth in the four areas over the 3 years of the program. Second, also annually starting after the first year, fellows complete annual reports during which they describe (in short answers) leadership growth, research progress, policy changes, dissemination of research, and challenges they have faced; challenges they have faced. Third and finally, after every in-person meeting we field a survey asking fellows to evaluate the experience generally and the specific sessions they attended. Members of the NPC leadership team (Leadership Contacts) also check in with their assigned team on an annual basis to assess progress and seek feedback on how the program can better serve fellows' needs. Future analyses by the NPC will describe the results of these data.

## Discussion of Practical Implications, Constraints, and Lessons Learned

As noted above, we have a wealth of data that we are analyzing for future reporting, but we have also generated the following *informal* or anecdotal lessons around the curriculum to date, as confirmed through consistent dialogue across program leadership, staff, partners, and fellows. These are both areas of learning and recognition of the constraints the program faces in the current cultural, social, and political context surrounding health equity research.

1. Adult learners require significant flexibility and adaptability in the curriculum, with fellows varying in how much experience they already have in each of the four curriculum domains. Further, there is great variation in which curricular elements fellows find most useful and essential to their progress in both research and leadership; there is no “one size fits all.”
2. The curriculum benefited from feedback and ability to adapt. The four curriculum pillars have remained in place throughout the program's first 5 years, yet each domain evolved and underwent revisions annually based on the needs of and feedback from fellows (e.g., push for more interactive learning experiences, draw on internal IRL vs. external experts, draw from more trainers and facilitators of color, etc.). From the beginning, the program adopted a “build the plane as we fly it” mentality, which allowed for rapid response to evaluation data.
3. Building social networks and social relationships with one another—as a key element of the IRL program experience—is core to the program's value as well as the transformative change that fellows experience. The curriculum and meetings provide the “glue” that regularly brings fellows together, and provides a common language and opportunities to interact, but their relationship building happens through strategic attention to relational leadership values as well as structuring open time for fellows to engage, network, and relax together.
4. Ensuring teams are grounded in their collaborative relationships and team values, are addressing conflict early, and are protected against burnout are foundational curriculum elements. Burnout among the leaders was a concern, beginning with the political dynamic in the United States initiated with the start of the Trump administration in 2016 which fellows experienced as hostile to health equity, and was maintained through the COVID pandemic and racial reckoning of 2020 and into 2021. Ongoing support for teams' collaboration and fellows' circumstances—and modeling this caring by the NPC team and staff—turned out to be an important aspect of the program experience.
5. While equitable collaboration is essential (as noted above), so too is preparing teams to be equitable in their community engagement activities. More curricular attention to the macro-level context of communities—including attention to power dynamics in communities, budgeting, engaging communities on study design, recruitment, analysis, dissemination planning and throughout community-focused translation of results, are critical domains of the curriculum on which to expand and build in future years. For instance, the Collaboration and Community Engagement curriculum evolved to build in direct and normalized interrogation of power dynamics in academic-community partnerships (38). Community-driven research partnerships—that embrace the principles of CBPR and yet go beyond them—honor cultural knowledge and practices, relationships, indigenous research methods, co-learning, community data ownership, and tangible benefits to the community from the research (39). In such partnerships, community power, wisdom, and leadership are not only acknowledged, but centered (40); these are among the central tenets of critical race theory (41).
6. Research partners and community partners have different needs with regard to the curriculum, and our most recent ongoing rapid evaluation and curriculum revisions are focused on how to ensure the value of the curriculum to the community leaders, as well as their networks.

## Conclusion

The Interdisciplinary Research Leaders program is a model for stepping outside of the traditional halls of research and developing evidence-based solutions in communities where those solutions can be most effectively applied. The focus on community engagement is a defining feature and one that underscores the importance of including individuals and the community contexts that are most impacted and often most knowledgeable about the threats to public health and health inequity. To achieve this, the program has also developed, and continues to develop, content and space for the fellows to practice equitable collaboration, community organizing, bridging relationships, and translating research into various contexts. The program also aims to enhance the leadership skills of researchers and community partners who will use these skills to continue their important work igniting action to advance health equity, well beyond their IRL fellowship (42). Finally, the community of practice that the program facilitates within the cohort and across cohorts is intended to be a lasting shared resource and evolving network to rely upon in collective efforts toward advancing a culture of health.

## Data Availability Statement

The original contributions presented in the study are included in the article/supplementary material, further inquiries can be directed to the corresponding author.

## Author Contributions

SG drafted the article. KC, JRM, BC, and ZB provided critical revisions of the article and SG incorporated all feedback. All authors participated in conceptual discussion of the curriculum and key components of the paper. All authors contributed to the article and approved the submitted version.

## Funding

Support for this article was provided by the Robert Wood Johnson Foundation (grant #78930).

## Author Disclaimer

The views expressed here do not necessarily reflect the views of the Robert Wood Johnson Foundation.

## Conflict of Interest

The authors declare that the research was conducted in the absence of any commercial or financial relationships that could be construed as a potential conflict of interest.

## Publisher's Note

All claims expressed in this article are solely those of the authors and do not necessarily represent those of their affiliated organizations, or those of the publisher, the editors and the reviewers. Any product that may be evaluated in this article, or claim that may be made by its manufacturer, is not guaranteed or endorsed by the publisher.
